# Progress in State Medicine

**Published:** 1900-12-29

**Authors:** 


### Progress in State Medicine.

**State Care of Consumptives.**—At Rutland,[1] Massachusetts, the first State Hospital for consumptive and tuberculous poor is now in working order. The trustees were appointed by the legislature, and a hundred and fifty thousand dollars voted toward the expenditure. A circular letter was sent to all the medical men in the state, and much valuable information was obtained. Rutland is situated eleven hundred and sixty feet above the sea, is sheltered by a hill to the north, which is a hundred feet higher, and included in the hospital grounds are two hundred acres of land. The wards are arranged like the spokes of a wheel, all leading at their northern end to a long corridor which connects them. At their southern end they are provided with a large covered glass solarium, and outside this a broad verandah, having three different exposures. The central building is two stories high and separates the male and female wards. The beds number one hundred and seventy-five. Internally all corners are rounded off, and the corridors have no stairs, but follow the natural grade. In each long ward there are twenty-one beds, and five additional rooms are provided, containing one or two beds for cases developing complications. The hospital is lighted throughout by electricity, and warmed by steam and hot air. Each patient is provided with a book, in which he enters the number of hours spent out of doors, condition of sleep, bowels and appetite. Pasteboard sputum-cups are given to each inmate for indoor use, and these are burnt at the end of the day. Away from the institution the patient expectorates into pieces of Japanese paper, which he puts into a self-closing rubber pouch, carried in the pocket. Three full and the three light meals are provided daily. Outdoor life is encouraged, and at night the ward windows are left open, in fact, in the winter the temperature has rarely risen above 40° F., day or night. No hopeless cases are admitted, and all cases not showing improvement at the end of six weeks, gauged by weight, temperature, cough, expectoration, are discharged. In the first six months of the hospital's existence 224 patients had already availed themselves of its benefits, a very large number of whom had been improved by an average stay of four months. Nathan Raw[2] speaking on rate-supported sanatoria for consumptives, emphasises the fact that tuberculosis is never directly hereditary, but in every case acquired. It is chiefly transmitted by the sputum of a consumptive to a previously healthy person, tuberculous meat, and milk from a tuberculous cow. The two latter sources of infection are being vigorously dealt with by the Medical Officers of Health. The danger lies in the first. Legislative or coercive measures of prevention would be futile until the public are thoroughly educated as to the cause, measures of prevention, and hope of cure. The upper and middle classes having learned this lesson will provide for themselves, the poor must be cared for at the expense of the rates. This is now being carried out in Liverpool. Representatives were chosen from the three large unions, aggregating nearly a million souls, and formed a committee, each union to bear its fair proportion of cost as allocated to it. The Local Government Board at once agreed to the scheme, and an excellent and easily accessible site has been selected on the river Dee, near Heswallon. The sanatorium is to be reserved for cases certified as early or easily curable. A man or woman cured of consumption is again a bread-winner, and not only himself but his wife and children removed from the rates. With regard to the working non-pauper class who could afford to pay something towards their maintenance, each County Council, Corporation, or District Council should provide sanatoria for their use. In a few years public opinion will be so awakened to the dangers of infection that a consumptive person will experience great difficulty in procuring employment, and thus become thrown upon the rates. The remedy for this is municipal sanatoria. Kenwood[3] advocates isolation hospitals for each district or group of districts, and where the medical officer and the medical attendant are prepared to certify that the isolation of the patient is the only measure by which others can be protected, power should be given and the means provided to secure such isolation. Very little has been done in the way of sanatoria for the poor. The general hospitals are getting disinclined to admit phthisical cases to the general wards, so that provision will have to be found for these cases, and the establishment of municipal sanatoria is certainly the best method. Woodcock[4] states that according to German statistics a sanatorium containing a hundred consumptives would cure absolutely ten to twelve of the hundred, and also almost cure another 20 per cent. of its patients. The sanatorium treatment for the working classes he thinks will hardly be a success at first, owing to the expense. There is great need of treatment and out-of-door life after leaving the sanatorium. Ransom[5] draws attention to the fact that in Germany the insurance companies, owing to the law allowing them to spend part of their funds in treatment in lieu of sick pay, have invested large sums in sanatoria. Statistics showed with one of the companies that out of 1,541 consumptives, 71 per cent. were restored to their working capacity, while in less than 7 per cent. of cases did the disease progress. The managers of the Poplar and Stepney Sick Asylum District[6] applied to the Local Government Board with regard to the building a hospital for the open-air treatment of their tuberculous poor. They had found on inquiry that in their own district alone there had been sixty-five cases of phthisis which might have been saved by this treatment. The Board was entirely in sympathy with the project, but suggested that a conference should be called of representatives of each poor-law authority in the metropolis, and provision made for the open-air treatment for the whole of the metropolis, the management to be under the control of some metropolitan authority. Acting on this, invitations were sent out,[7] and at the conference seventy delegates were present, representing twenty-six out of the thirty-two Metropolitan Boards of Guardians. It was unanimously decided that the time had arrived when some provision should be made for the treatment by the open-air method of the sick poor of the metropolis suffering from phthisis. A deputation was appointed to present the resolution to the President of the Local Government Board.

Morris[8] points out that measures of protection could be taken by the State, municipal and local sanitary authorities, against infection from infected persons, infected food-stuffs, and animals, and whilst agreeing with Lord Salisbury that the aid of the secular arm should not be invoked deprecates that the State should be content with maintaining an attitude of benevolent neutrality. We should at least be protected from the importation of tuberculous cattle from abroad. Municipal and local authorities could do much in the amelioration of the condition of the poor, in drainage of soil, cleansing of towns, preventing overcrowding, and the provision of healthy dwellings. Every town should have its own sanatorium. Its object should be twofold: (1) Isolation of those cases which would be a source of infection outside, and (2) a place where the best treatment would be provided for those who could not otherwise afford it.

[1] Med. Rec., Oct. 8, 1899. [2] Brit. Med. Jour., Aug. 18, 1900. [3] Ibid. [4] Brit. Med. Jour., Oct. 13, 1900. [5] Tuberculosis, Oct. 1900. [6] Lancet, Aug. 11, 1900. [7] Brit. Med. Jour., Oct. 20, 1900. [8] Brit. Med. Jour., Sept. 16, 1899.

